# Improvement of Mechanical Properties and Forming Efficiency during Hot Gas Forming of CFRP Curved Surface Components

**DOI:** 10.3390/ma14185316

**Published:** 2021-09-15

**Authors:** Yizhe Chen, Yi Lin, Hui Wang, Zhiwen Liu, Lin Hua

**Affiliations:** 1 Hubei Key Laboratory of Advanced Technology for Automotive Components, Wuhan University of Technology, Wuhan 430070, China; yzchen@whut.edu.cn (Y.C.); yilin01@whut.edu.cn (Y.L.); ydream@whut.edu.cn (Z.L.); hualin@whut.edu.cn (L.H.); 2 Hubei Collaborative Innovation Center for Automotive Components Technology, Wuhan 430070, China; 3 Hubei Engineering Research Center for Green & Precision Material Forming, Wuhan 430070, China

**Keywords:** carbon fiber reinforced plastics, curved surface components, hot gas forming process, defect control, mechanical properties, mechanism analysis, forming efficiency

## Abstract

Carbon fiber reinforced plastics (CFRP) are widely used in aerospace and new energy vehicles due to their high specific strength and flexible design ability. At present, the traditional forming process of CFRP curved surface components has problems of low mechanical properties and long processing time. In this paper, a new method of hot gas forming was proposed to obtain CFRP components. By applying high temperature and high-pressure gas on one side of CFRP, the material was forced to deform and solidify at the same time. A special device for hot gas forming was designed and developed. The curing behavior and mechanical properties of original CFRP plates were studied. The main defects and the corresponding control methods of hot gas forming parts were analyzed by forming spherical parts, and the feasibility of the hot gas forming process was verified. Taking the battery cover plate of a new energy vehicle as the research object, the influence of forming temperature, gas pressure, pressurization rate and other process parameters on the mechanical properties of complex CFRP components were analyzed. The mechanism of both strength and efficiency improvement was analyzed. The results showed that with the increasing of gas pressure, the tensile strength and forming efficiency of the CFRP curved components were improved obviously. Under reasonable forming parameters, the tensile strength of the obtained parts was increased by 37%, and the forming efficiency was increased by 58%. The fiber bundles were distributed more evenly and compactly under the hot gas forming. This showed that the use of hot gas forming had good potential in the preparation of high-performance CFRP parts, which was helpful to improve the processing efficiency and forming quality of CFRP curved parts in the aerospace and new energy automotive fields.

## 1. Introduction

In recent years, light materials such as aluminum alloy [1], magnesium alloy [2], titanium alloy [3] and fiber composite materials [4] have been widely used in various manufacturing industries. Due to the characteristics of high strength, high rigidity, low density, and convenience of design, carbon fiber reinforced plastics (CFRP) materials have been widely used in aerospace, new energy vehicles and advanced sports equipment. Common forming methods for complex parts of CFRP materials include autoclave molding [5], resin transfer molding [6], and hot stamping [7].

Autoclave molding is mainly used in the aerospace and aviation fields for high precision and high-quality composite structures. There is sufficient space in the tank to be used for processing and forming large products and multiple products simultaneously. This method has the advantages of uniform distribution of pressure and temperature and uniform structural properties of the formed components [8]. The high-pressure process of autoclave molding facilitates the infiltration of the resin in the CFRP and reduces the porosity in the parts. The porosity of the components will significantly reduce its mechanical properties [9]. The voids of parts will lead to premature failure of loaded materials, which affects the dynamic and steady response of materials [10]. In addition, the high curing pressure in the autoclave increases the volume fraction of fiber in the parts and improves the mechanical properties [11]. However, the autoclave molding process has problems of high processing cost and long forming time. The resin transfer molding (RTM) process has a wide range of applicability. With the increasing use of fiber reinforced thermosetting composites and the demand for productivity and cost, the resin transfer molding (RTM) process has become a priority research field of mass production [12]. Chen et al. [13] prepared CFRP laminates by the RTM process and analyzed the impact damage responses of carbon fiber reinforced polymer (CFRP). Its influence on the compression mechanical responses of CFRP laminates was also analyzed. Compared with the autoclave molding process, the RTM process has the advantages of short molding cycle and low manufacturing cost. It can manufacture parts with complex shapes. However, after the resin is injected, a longer filling time is required to make the resin flow and infiltrate uniformly. Therefore, the production efficiency was low. In the RTM process, resin is injected into the mold, which tends to lead to uneven injection of resin, resulting in poor quality of the formed parts.

Compared with the autoclave process and the RTM process, the hot stamping process is more efficient [14]. In order to reduce manufacturing costs, the research on hot stamping of metals [15] and composite materials has become a hot topic. Uriya [16] used continuous carbon fiber reinforced polypropylene composite materials to manufacture parts with complex surface contours. Their work proved that hot stamping can be used for various special-shaped parts. Sun et al. [17] conducted hot stamping experiments on carbon fiber reinforced polypropylene (CF/PP) materials and made curved surface components. This study confirmed that hot stamping can be used to stamp complex geometry parts. However, the heating and deformation process of these methods were carried out separately. The sample needs to be heated in a heating furnace and then placed on the mold for stamping. The whole process was tedious, which made long forming cycles and low forming efficiency. Meanwhile, it was difficult to position the heated sample. The temperature of the place where the sample was in direct contact would drop rapidly. It would cause uneven heating, which reduced the final performance of the sample.

Hot gas forming technology is often used in the processing of metal materials [18]. The emergence of hot gas metal forming technology was initially to develop a lower cost tubular metal forming process [19]. Hot gas forming technology is a high-temperature metal forming technology developed on the basis of traditional hydraulic forming and superplastic forming [20]. Botashev et al. [21] studied the hot metal gas forming and concluded that the key parameters which affect the forming are temperature and pressure. Wang [22] studied the hot gas pressure forming (HGPF) of Ti-55 high temperature titanium alloy. Tang [23] et al. made a V-shaped trough containing deep uneven concavities using superplastic AA5083 by using HMGF technology. With the research on the HMGF process, it is found that the process has the advantages of high processing flexibility, low mold cost, and short manufacturing cycle [24]. The large number of applications in the field of sheet metal forming provides a reference for the preparation of composite materials.

In order to solve the problems of long processing cycle and poor performance of traditional methods, this paper proposed a hot gas forming process of CFRP complex parts. By applying high temperature and high-pressure gas on one side of CFRP, the material was forced to deform and solidify at the same time. A special device for hot gas forming was designed and developed. The main defects and corresponding control methods of hot gas forming parts were analyzed by forming spherical parts, and the feasibility of the hot gas forming process was verified. Taking the battery cover plate of a new energy vehicle as the research object, the influence of forming temperature, gas pressure, pressurization rate and other process parameters on the mechanical properties of complex surface components were analyzed.

## 2. Experimental

### 2.1. Hot Gas Forming Process

**Figure 1:**
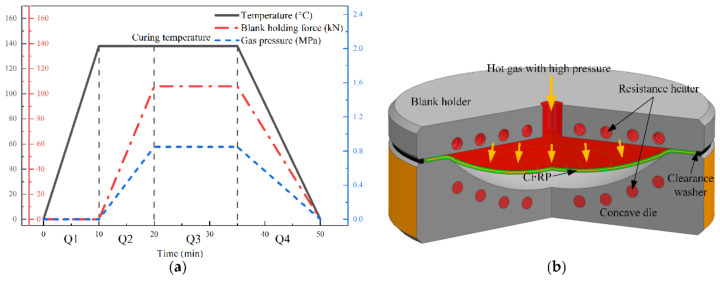
Principle of hot gas forming: (**a**) variation of main parameters; (**b**) forming mold and process.

As shown in Figure 1, the hot gas forming device mainly consists of upper die, lower die, resistance heater, gas pipeline and other parts. The process parameters depend on the type and performance of CFRP. The prepreg was thawed at room temperature for 1 hour before lamination. CFRP was laminated by 10 layers of prepreg. For each layer of prepreg, the surface was repeatedly squeezed with a hard roller. After the lamination, the prepreg was coated with a breather on its surface and placed in a vacuum bag. After the vacuum degree reached 0.08 MPa, the prepreg was taken out and ready for the hot gas forming. This progress could minimize air retention between layers during lamination.

The steps of the hot gas forming process and the variation trend of process parameters were shown in Figure 1a. The whole process consisted of four steps, Q1–Q4. In step Q1, the mold was heated to the forming temperature of the CFRP. In step Q2, the CFRP was laid on the lower die with silicone membrane on its surface. This method could keep the carbon fiber from being damaged and avoid the infiltration of the air. Next, the upper and lower dies were clamped together under a certain force. Meanwhile, the CFRP was heated to the resin curing temperature according to its characteristics, and high-pressure gas was led in to deform the CFRP. The gas used in the process was compressed air. In step Q3, the temperature and pressure of the mold remained constant after forming to ensure the CFRP solidifying process. Finally, in step Q4 the process of pressure relief, cooling and demolding was carried out.

**Figure 2:**
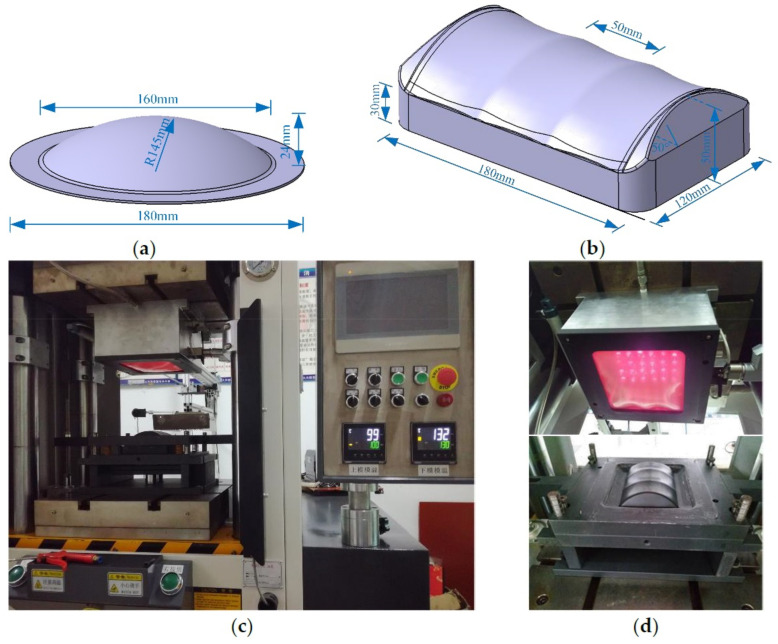
Geometrical model of forming parts and mold: (**a**) spherical simple part; (**b**) complex part; (**c**) forming machine; (**d**) forming mold.

In this research, two kinds of parts were used to carry out the hot gas forming process experiment. As shown in Figure 2a, the size of the spherical part was 145 mm, and the spherical depth was 24 mm. The common defects of the hot gas forming process were analyzed by forming this simple spherical part to verify its feasibility. On this basis, taking the battery cover plate of new energy vehicle as the research object to carry out the hot gas forming process. Figure 2b showed the three-dimensional model of the battery cover plate. The size of the model was 180 mm × 120 mm × 30 mm, and the two sides of the mold core formed a 50° angle. There were three concave arc surfaces in the middle with irregular shape. Figure 2c and d showed the experimental equipment and mold in this paper. Forming temperature, gas pressure, pressurization rate could be adjusted independently. The maximum pressure could reach 30 MPa. During the hot gas forming process, high-temperature and high-pressure gas were used as the pressure transmission medium. Only a single-side mold was needed. The upper of the mold was a cavity. The bottom was sealed with a silicone film to ensure the sealing of high-pressure gas when the mold was clamped and pressurized. The lower mold could be taken out for easy replacement.

Firstly, the feasibility of the hot gas forming process was studied. Then, the spherical parts were prepared using the hot gas forming process. Next, the defects of spherical parts were analyzed and solved by adjusting the forming process.

Subsequently, the battery cover plate was taken as the research object. The analysis of the influence of forming temperature, gas pressure, pressurization rate on performance was focused on. Among them, the gas pressure was 0.4, 0.7, 0.9 MPa and the pressurization rate was 0.005, 0.01, 0.1 MPa/s.

### 2.2. Traditional Forming Process

The traditional forming process in the experiment was the common manual laminating. The specific process was shown below. First, the prepreg was cut to the required size, and then soften by being thawed at room temperature for 1 hour. Before lamination, the release agent was applied to the mold. The thawed and softened prepreg was laid on the mold layer by layer, during which the surface was pressed with a hard roller to squeeze out the air. After the lamination, the part was vacuum bagged. An unperforated release film was applied to the laminate. The breather was used on the reverse of the mold. Then, the vacuum pump was used to extract the air in the vacuum bag. After the above steps, the prepreg was placed in the oven for heating. In order to study the difference of performance and forming efficiency between hot gas forming and traditional forming, the forming temperature was the same as those used in the hot gas forming process.

### 2.3. Tensile Test

**Figure 3:**
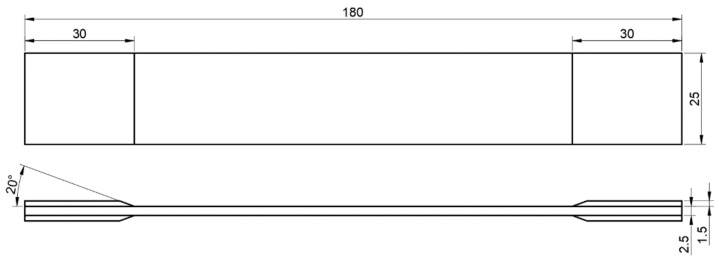
Specimen size of CFRP material for tensile test.

The mechanical properties of CFRP were obtained using tensile tests. The tensile test of the composite material was carried out according to GB/T 3354-2014 “Test Method for Tensile Properties of Oriented Fiber Reinforced Polymer Matrix Composites”. The size of the tensile samples is shown in Figure 3.

### 2.4. Observation of Morphology

In order to explore the micro-morphology of parts under different process parameters and analyze the mechanism of tensile strength variation, the fracture morphology of tensile samples was observed with an Olympus optical microscope DSX510 manufactured by Olympus Corporation (Beijing, China) Co., Ltd.

## 3. Curing Behavior and Mechanical Properties of CFRP

A carbon fiber/epoxy composite was used in this study, in which the resin content was 42% and the thickness was 0.3 mm. It was produced by Weihai Guangwei Composites (Weihai, Shandong, China) Co., Ltd. The epoxy resin was R5600W3K, and it had the best fluidity at 130 °C. The carbon fiber was SYT35/3K. Its specific parameters were shown in Table 1.

**Figure 4:**
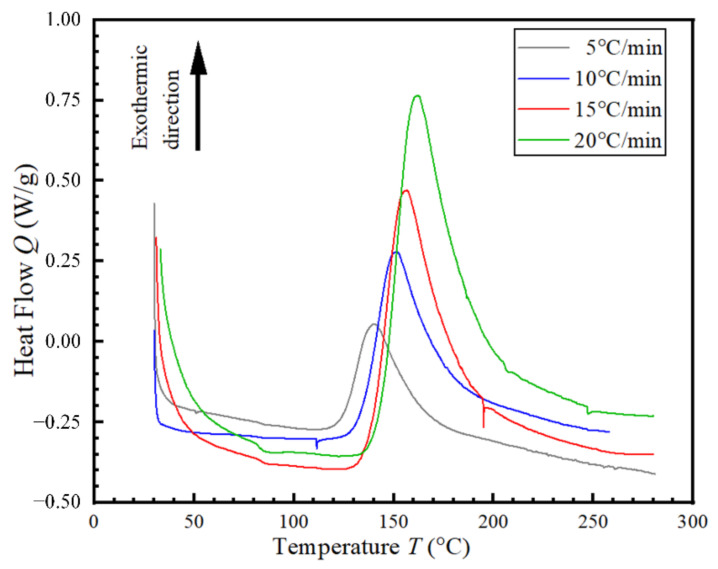
Heat flow of CFRP non-isothermal curves under various heating rates.

The curing behavior of the CFRP was tested by TA-DSC250 differential scanning calorimetry. The effects of different temperatures on the curing reaction of the CFRP under atmospheric pressure were studied. Firstly, the non-isothermal DSC test was performed at heating rates of 5, 10, 15, and 20 °C/min respectively. The obtained curves are shown in Figure 4.

From Figure 4, it can be found that the heat flow of the four heating rates decreased rapidly before the temperature of 50 °C. With the increase in the heating rates, decrease rates of the heat flow slightly slow down. The decrease of initial heat flow was due to the endothermic reaction of CFRP. The faster the temperature rose, the slower the heat flow dropped. The reason was that the rapid temperature change led to the shorter heat absorption time of the CFRP. Higher temperature was needed to absorb enough heat to reach the steady state. Between 50 and 120 °C, the heat flow slowly decreased and gradually flattened out. At this time, the CFRP had absorbed enough heat. Therefore, the resin gradually reached a steady state. There was a fluctuation around 110 °C under the heating rate of 10 °C/min, which was speculated that there were subtle impurities inside. When the temperature reached 120 °C, the heat flow of the four heating rates began to increase rapidly and monotonously. If the temperature continued to rise, the resin began to release excess heat. Thus, the heat flow gradually increased. The faster the temperature rose, the faster the heat was released, and the more heat was released. Peak values of heat flow can be obtained between 140–160 °C. In addition, an increased heating rate resulted in higher peak value. After reaching the peak value, the heat flow gradually decreased and finally tended to constant. Then, the temperature started to exceed the optimal range of resin curing, and the heat release of resin began to slow down. The heat flow began to decrease gradually. The heat release ended after the curing was complete.

**Figure 5:**
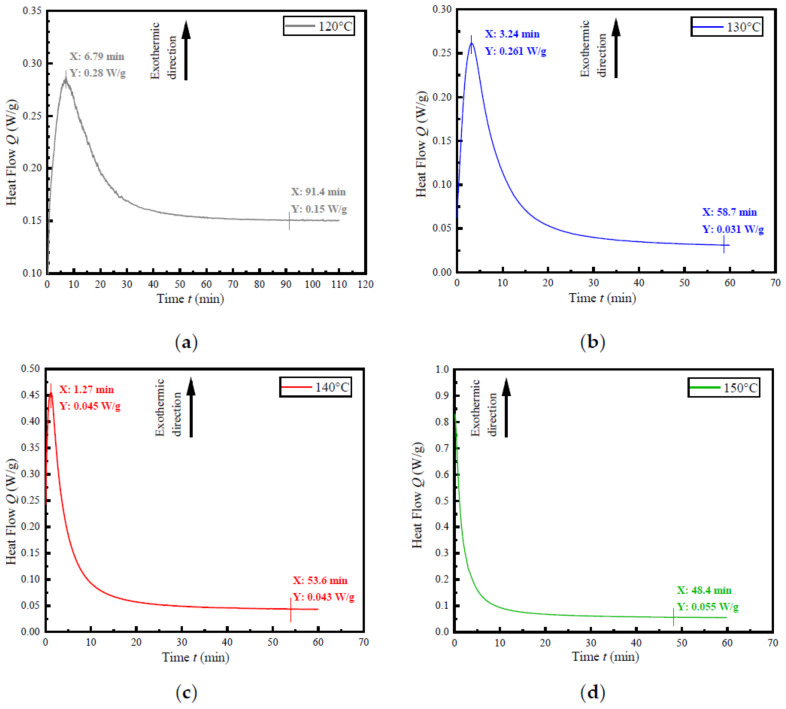
Isothermal DSC curves at different temperatures: (**a**) 120 °C; (**b**) 130 °C; (**c**) 140 °C; (**d**) 150 °C.

According to the calculation of DSC test results of four different heating rates, the average initial curing temperature of the resin was 133.5 °C. Since the initial curing temperatures of the four heating rates were all between 120–150 °C, isothermal DSC tests at 120, 130, 140 and 150 °C were carried out respectively to obtain the time required for the CFRP. The results were shown in Figure 5.

The results showed that with the increase in curing temperature, the peak value moved forward, which indicated the accelerated curing process. According to the change of specific heat in the test, the curing time of CFRP at different temperatures could be predicted. It can be seen from Figure 5d that the curing time of CFRP was the shortest at the highest temperature (150 °C), which was 48.4 min. While it took 91.4 min to complete the curing at 120 °C.

According to previous analysis, the mechanical tests of CFRP original plates were carried out. The curing temperature was 133.5 °C and the heating time was 60 min. According to the standard in Section 2, the tensile strength of three groups of CFRP samples were 237, 240 and 249 MPa, with an average of 242 MPa.

It can be seen that during the above experiments, the curing of CFRP under atmospheric pressure not only had low strength, but also had low production efficiency. The use of hot gas forming has an impact on mechanical properties and forming efficiency of CFRP parts is the focus of the study. Therefore, combined with the curing curve of DSC test and the fluidity of the resin, the curing temperature range of the hot gas forming process was set at 120–140 °C.

## 4. Feasibility Analysis about Hot Gas Forming of CFRP Components

The hot gas forming process has been widely used in the metal forming area, but the feasibility of hot gas forming on CFRP still needs to be confirmed. Therefore, spherical CFRP parts were obtained using the mentioned device. Several kinds of defects were found during the forming process. Suitable control strategy was given to suppress these defects and the feasibility of hot gas forming of CFRP was verified.

### 4.1. Typical Defects

**Figure 6:**
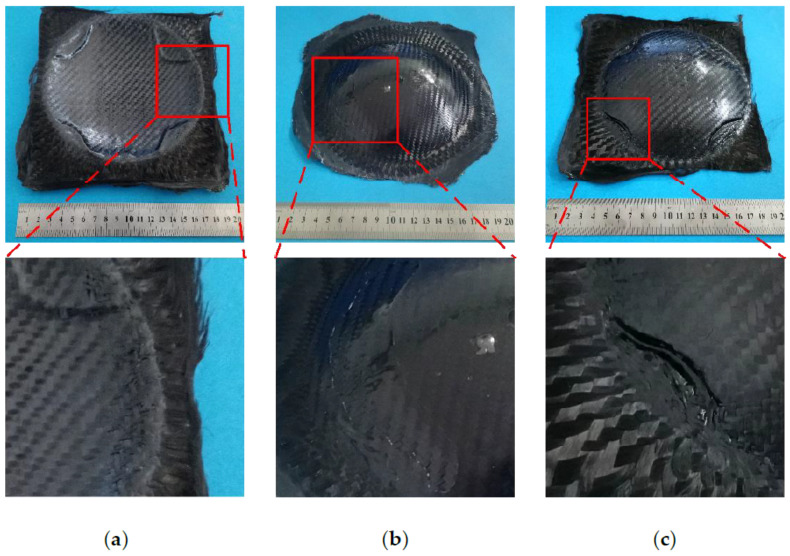
Three types of forming defects: (**a**) crack; (**b**) resin surplus; (**c**) wrinkling.

During the hot gas forming process, some defects including cracks can be found, as shown in Figure 6. The forming process parameters of the part shown in Figure 6a were: temperature of 120 °C, blank holder force of 200 kN, gas pressure of 0.7 MPa, and pressurization rate of 0.01 MPa/s. According to the measurement, the slip of CFRP was 11.5 mm in both vertical and horizontal directions. Some crack could be seen at the surface parts of CFRP, which were distributed in the circular corner. Cracks of carbon fiber in the corner area would reduce the stiffness of spherical parts, weaken the mechanical properties and shorten the service life of parts. The main reason of cracks was that in the process of forming, the blank holder force was too large at 200 kN, the carbon fiber was stretched under the force of blank holder and gas pressure. The carbon fiber in the forming corner area became more dispersed, leading to the weakening of the carrying capacity in this area. Under the continuous action of blank holder force and gas pressure, part of the fiber reached the strength limit and brittle fracture occurred.

It can be seen from Figure 6b, resin surplus appeared in the arc area of the curved parts. The forming process parameters of the part shown in Figure 6b were: temperature of 140 °C, gas pressure of 0.4 MPa, and pressurization rate of 0.1 MPa/s. According to the measurement, the slip of CFRP was 12.6 mm in both vertical and horizontal directions. Resin surplus not only resulted in rough surface and poor surface quality in the arc area, but also led to resin deficiency and dry spot in other areas of spherical parts. These problems would seriously affect the forming quality and reduce the service life of parts. The main reason of resin surplus was that the resin matrix melted when heated and its viscosity decreased. It flowed downward and aggregated in the arc surface area of CFRP curved part under the action of gravity and gas pressure.

It can be seen from Figure 6c, the wrinkling appeared in the circular corner of the curved components. The forming process parameters of the part shown in Figure 6c were: temperature of 130 °C, gas pressure of 0.9 MPa, and pressurization rate of 0.005 MPa/s. According to the measurement, the slip of CFRP was 11.9 mm in both vertical and horizontal directions. Slight wrinkling would affect the surface quality and forming accuracy of the component. Severe wrinkling would reduce the stiffness and strength of the composite material and significantly affect its performance. When the blank was round, the wrinkling area was mainly in the arc and corner area. As carbon fiber almost had no plastic deformation, wrinkling was a common forming defect in CFRP parts. It was the unexhausted gas between the blank and mold that caused the wrinkling. The main reason was the small center tension of the blank. The blank was prematurely attached to the concave mold surface under the action of gas pressure, blocking the vent holes. This problem caused the gas between the blank and the mold not be discharged smoothly during the forming process.

### 4.2. Control Methods of Defects and Process Feasibility

**Figure 7:**
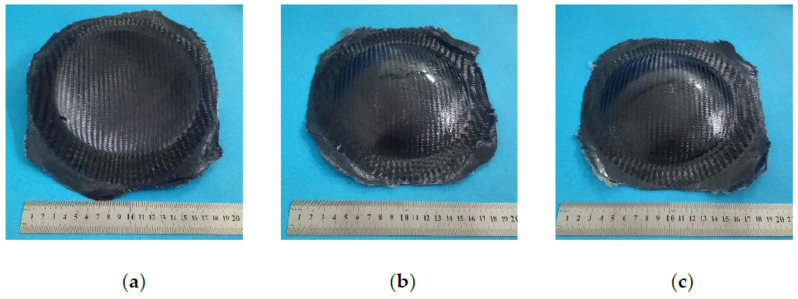
Well-deformed parts after defect control: (**a**) crack control; (**b**) surplus control; (**c**) wrinkling control.

In order to prevent the fiber from being stretched, the blank holder force was appropriately reduced to 100 kN, and the fiber lay-up was modified to improve the fiber resistance ability. The consistency of the fiber direction between the layers of CFRP was strictly ensured when the CFRP was subsequently laminated. As shown in Figure 7a, the change of the lay-up method enhanced the strength of the corner area of the carbon fiber and strengthened its ability to resist deformation and bending. Therefore, the crack defect can be suppressed.

It can be found that the phenomena of resin melting and surplus occurred in the initial heat preservation stage of the entire heat process. Therefore, the heat process method was improved. The preheating and insulation stage of CFRP was skipped, and high-pressure gas was passed to press the blank during heating. It would weaken the effect of the gravity of the resin itself and avoid the resin aggregation caused by the long-term downward flow of the resin. The improvement effect of formed part was shown in Figure 7b.

Through the wrinkling analysis in Section 4.1, it was the excessive blank holder force that made the gas discharged when forming. Due to the good sealing performance and elasticity of the silicone membrane, the upper and lower silicone membranes formed a sealing space. Therefore, in order to further solve the wrinkling defect, the appropriate blank holding force was adjusted to 100 kN through multiple experiments to solve the wrinkling problem. The improvement was shown in Figure 7c.

After solving the above-mentioned defects, the study showed that the hot gas forming process was feasible for the preparation of CFRP.

## 5. Deformation Behavior of CFRP Complex Curved Components in Hot Gas Forming

### 5.1. Trial Production of Complex Curved Surface Components

**Figure 8:**
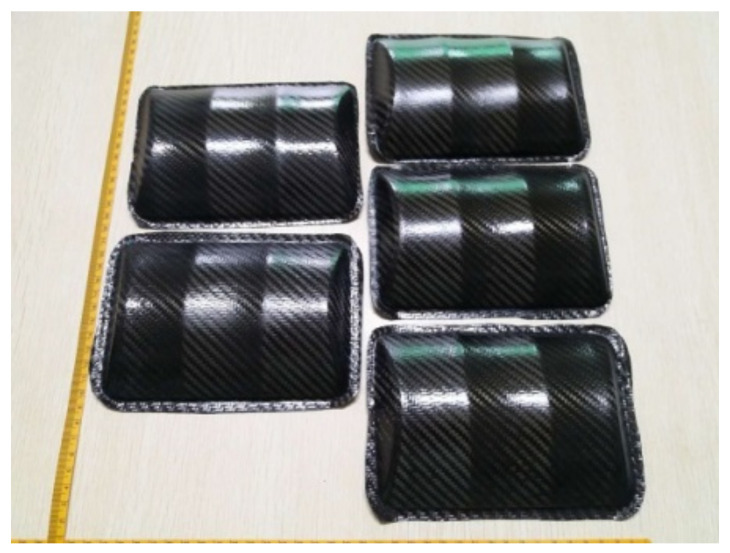
Complex CFRP components with no defects using hot gas forming.

Using the similar modifying strategy of forming parameters in the previous section, the trial production of complex CFRP components using hot gas forming industry was further considered. Figure 8 showed some of the components obtained by the hot gas forming process. It can be seen that the forming effects including forming precision and surface quality were great with no obvious defects.

### 5.2. Variation of Mechanical Properties and Forming Efficiency

**Figure 9:**
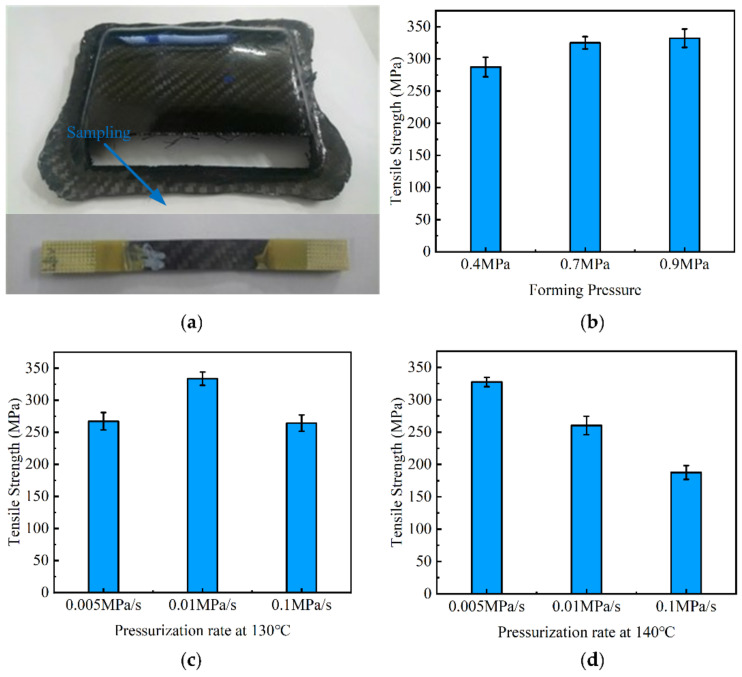
Tensile test of complex CFRP components: (**a**) location of sample; (**b**) influence of forming pressure; (**c**) influence of pressurization rate at 130 °C; (**d**) influence of pressurization rate at 140 °C.

In order to obtain the influence of forming parameters on the mechanical properties, several tensile tests were carried out. The location of tensile sample and corresponding results could be found in Figure 9. In order to maintain high efficiency of the hot gas forming, the total time of pressurization and pressure holding was kept constant at different temperature. Considering the properties of the resin in the prepreg, the pressure holding time was controlled at least 30 min. The pressurization process also took several minutes, therefore, the total time of pressurization and pressure holding was controlled to 35 min. The experiment result showed that CFRP could not be cured within 35 min at 120 °C, and the forming quality of the obtained part was poor. It could not be effectively tested for strength. In order to study the strength of efficient forming of parts, the result of 120 °C was excluded.

Effect of pressure on the tensile properties of CFRP was studied by keeping the forming temperature and pressurization rate unchanged during the experiment. As shown in Figure 9b, the tensile strength of the corresponding pressure was 287.4, 325.2 and 332.3 MPa at 130 °C. The results showed that the tensile strength of CFRP gradually increased with the increase in forming pressure. The tensile strength increased by about 15% with the pressure increased from 0.4 to 0.9 MPa. Compared with the strength of traditional forming CFRP, the strength was increased about 37%.

Effect of pressurization rate on the tensile properties of CFRP was studied by keeping the forming temperature and gas pressure constant. As shown in Figure 9c, at different pressurization rates, the tensile strengths of the corresponding specimens were 267.4, 333.6 and 264.3 MPa at 130 °C. The results showed that, under the temperature of 130 °C, the tensile strength of CFRP would gradually increase with the increase in the pressurization rate, but that the strength would decrease after reaching a peak.

The effect of the forming temperature on the tensile properties of CFRP was studied by keeping the forming pressure and pressurization rate constant. By comparing Figure 9c,d, the tensile strength of the corresponding specimens at 140 °C was 327.6, 260.3 and 187.6 MPa. The results showed that, under the temperature of 140 °C, the tensile strength of CFRP gradually decreased with the increase in the pressurization rate.

**Figure 10:**
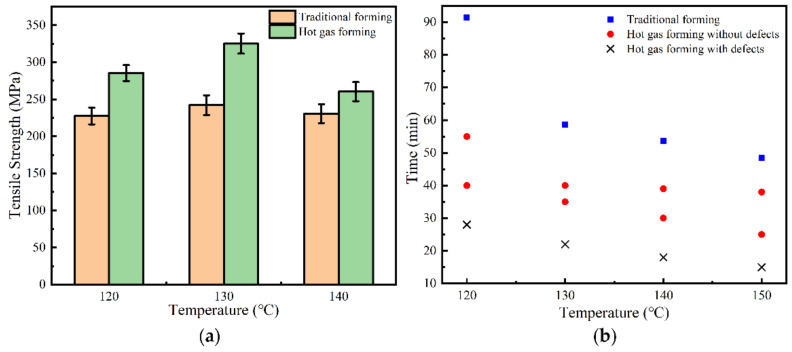
Comparison results of traditional process and hot gas forming process: (**a**) tensile strength; (**b**) forming efficiency.

Compared with the tensile strength and forming time under the traditional forming process, the hot gas forming process had higher performance and forming efficiency, as shown in Figure 10. From the results, it can be observed that the tensile strength of the CFRP made by the hot gas forming process was increased by 26% at 120 °C, 34% at 130 °C and 13% at 140 °C. The forming efficiency was improved by 92% at 120 °C, 57% at 130 °C, 55% at 140 °C and 54% at 150 °C. The experimental results showed that the hot gas forming process had a good potential for the preparation of high-performance CFRP parts, which was helpful to improve the forming quality and processing efficiency of CFRP complex surface components.

### 5.3. Mechanism Analysis of Enhanced Properties

**Figure 11:**
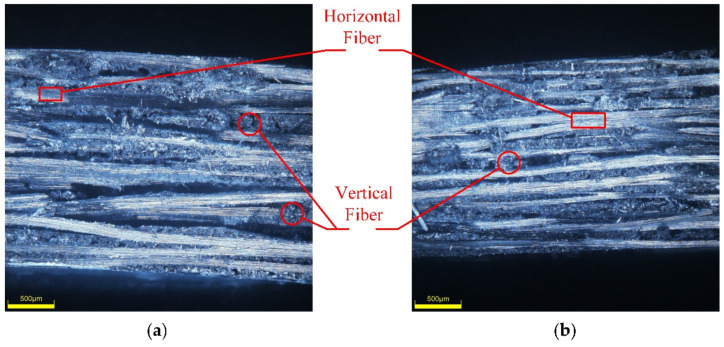
SEM images of complex CFRP components under gas pressure of (**a**) 0.4 MPa; (**b**) 0.9 MPa.

The prepreg used in this experiment was orthogonal braided. Therefore, the fibers had two directions. In Figure 11, there were two kinds of areas: highlighted areas and dark areas. The highlighted areas showed the horizonal direction of fibers. The dark areas showed the vertical direction of fibers. As shown in Figure 11, the thickness of the curved component under 0.4 MPa was 2.3 mm, while the thickness of the curved component under 0.9 MPa was 2.0 mm. The wall thickness decreased by 13.4% after the pressure increased. The increase in gas pressure accelerated the flow of materials in the mold cavity, made the mold and the material fit closely. In the tensile test of CFRP, carbon fiber was the main load-bearing material. With the increase in gas pressure, the compactness of the material was enhanced. The interlayer spacing inside the material were reduced. As a result, the volume fraction of carbon fiber in the whole composite was increased. Therefore, with the increase in gas pressure, the thickness of curved components would decrease due to the decrease in voids. According to the Equation (1) of tensile strength, the tensile strength was inversely proportional to the thickness of curved surface components. The strength of curved surface components would increase with the increase in gas pressure.

(1) $$\sigma =\frac{F_{b}}{dh}$$

**Figure 12:**
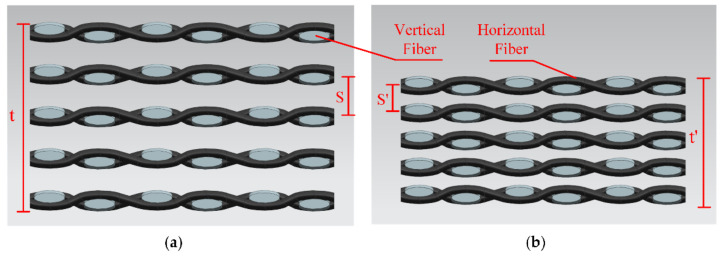
Schematic diagram about the influence of gas pressure on thickness (**a**) 0.4 MPa; (**b**) 0.9 MPa.

The fiber distribution diagram under two kinds of gas pressures is shown in Figure 12. It can be observed that when the pressure increased, not only the plate thickness decreased to t’, but also the fiber distance decreased with the increase in pressure. It can be seen that S’ was less than S, the fiber distribution was closer, and the total tensile strength was improved.

**Figure 13:**
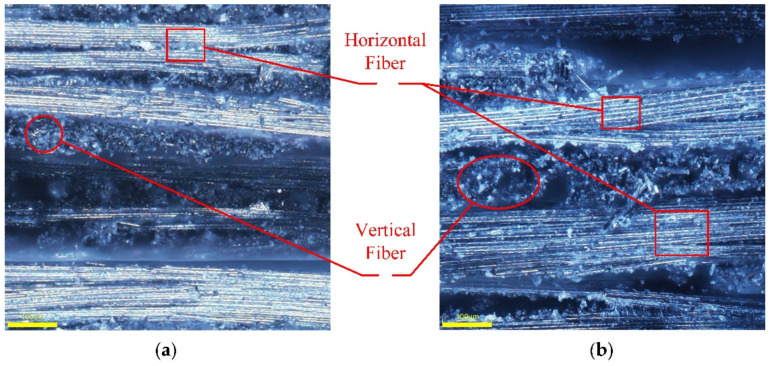
SEM images of complex CFRP components under pressurization rate of (**a**) 0.1 MPa/s; (**b**) 0.01 MPa/s.

**Figure 14:**
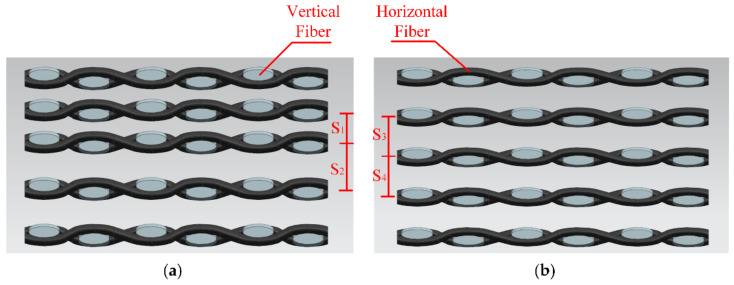
Schematic diagram about the influence of pressurization rate on thickness (**a**) 0.1 MPa/s; (**b**) 0.01 MPa/s.

It can be seen from Figure 13, that when the pressurization rate was too fast at 0.1 MPa/s, it would result in the enrichment of the upper fiber. The distribution was uneven, which led to poor tensile properties. As shown in Figure 14a, the distance S2 was greater than S1. When the pressurization rate was too fast, the fiber was densely distributed in the upper part and sparsely distributed in the lower part. This problem made the tensile properties worse. In Figure 14b S3~S4, the fiber was evenly distributed, which made the tensile properties stronger. The pressurization rate would affect the deformation rate of CFRP curved surface components. It can be seen from Figure 9c that the tensile strength increased first and then decreased with the decrease in pressurization rate at 130 °C. If the pressurization rate was too high, the carbon fiber would deform and fit prematurely. At this time, the flowing of resin was not enough, which made the infiltration uneven and reduced the mechanical properties. Proper reduction of the pressurization rate effectively ensured the full infiltration of the epoxy resin and made the deformation process of carbon fiber synchronize with the curing process of the resin. The interfacial compatibility of the fiber and resin was improved, and the fiber and resin were evenly distributed.

In order to study the influence of gas pressure, pressurization rate and forming temperature on the hot gas forming process, the total forming time was kept unchanged. If the pressurization rate was too low, it took more time to reach the required pressure. Therefore, the pressure holding time will be shortened. From the DSC test in Figure 5, it can be seen that the curing time of the resin at 130 °C was longer than at 140 °C. Therefore, unlike 140 °C, a slightly faster pressurization rate at 0.01 MPa/s was required to provide sufficient pressure holding time at 130 °C.

The resin had low fluidity at the temperature of 120 °C; the penetration of resin in the carbon fiber was not enough, and the properties of the carbon fiber were not fully developed. Therefore, hot gas forming of CFRP requires a higher forming temperature. Based on the DSC test curve in Figure 5, the curing time of the resin decreased significantly with the increase in the temperature. The curing reaction of the resin was relatively rapid at 140 °C. Therefore, it did not need too much time for pressure holding at 140 °C. The tensile strength increased continually with the decrease in the pressurization rate at 140 °C. A lower pressurization rate was required to ensure sufficient crosslinking and equidistribution of resin. This method could get higher tensile strength.

## 6. Conclusions

In this paper, the hot gas forming process of CFRP is proposed to improve the forming quality. The specific conclusions are drawn as follows:

1. The curing behavior and mechanical properties of CFRP were analyzed. The DSC test of CFRP showed that its curing temperature was about 133.5 °C. The high temperature curing experiment and performance test of CFRP were carried out. It was found that the forming efficiency and performance of CFRP were low. The shortest curing time was 48.4 min according to the DSC test, and the tensile strength obtained by traditional forming was 242 MPa.
2. A new method of hot gas forming was proposed to obtain CFRP components. A special device for hot gas forming was designed and developed. A simple spherical part was formed by the hot gas forming process. Cracking, resin surplus and wrinkling, three defects that occurred in hot gas forming of CFRP, were summarized. Under the suitable forming parameters, the defects were controlled well, and the feasibility of the process was verified.
3. Taking the battery cover plate of a new energy vehicle as the research object, the hot gas forming of complex curved components was carried out. The results showed that compared with the traditional process, the forming performance and efficiency of the hot gas forming process were improved by 37% and 58%. With the increase in gas pressure, the compactness of the curved components was improved. Its strength was increased by about 40 MPa. When the forming temperature was 130 °C, it had better forming performance. Its tensile strength reached 288.4 MPa, which was increased by about 11%. With the increase in pressurization rate, the tensile properties at 130 °C first increased and then decreased, and reached the maximum value of 333.6 at 0.01 MPa/s.
4. The enhancement mechanisms of the forming temperature, gas pressure and pressurization rate on the tensile properties of the parts were analyzed. With the increase in gas pressure, the carbon fiber bundles in the CFRP were bonded more closely, and the compactness of the curved components was improved. Appropriate pressurization rate made the carbon fiber bundles evenly distributed in the curing process and improved the forming performance of the CFRP. The proper forming temperature and the correct pressurization rate provided enough reaction time for the crosslinking curing of the resin, thus improving the strength of the CFRP.

## Figures and Tables

**Table 1:** Specific parameters of carbon fiber.

Tensile Strength	Elastic Modulus	Elongation
3.5 GPa	230 GPa	1.5%
**Linear Density**	**Monofilament Diameter**	-
1.78 g/cm^3^	7 μm	-

## Data Availability

Data is contained within the article and can be requested from the corresponding author.
